# The Year of the Nurse during the COVID-19 Pandemic

**DOI:** 10.3390/nursrep11040071

**Published:** 2021-09-29

**Authors:** Anna Bartosiewicz, Kinga Harpula, Edyta Łuszczki

**Affiliations:** 1 Institute of Health Sciences, Medical College of Rzeszów University, 35-959 Rzeszów, Poland; eluszczki@ur.edu.pl; 2 Medical College, University of Information Technology and Management in Rzeszów, 35-225 Rzeszów, Poland; wilkinga@gmail.com; 3 Healthcare Complex No. 2, Specialist Outpatient Clinic, Diagnostic Center, 35-005 Rzeszów, Poland

**Keywords:** nurse, Year of the Nurse, COVID-19 pandemic

## Abstract

The year 2020 was established by the World Health Organization as The Year of the Nurse and Midwife to emphasize the importance of this profession to the healthcare system. Strange but true, nurses around the world celebrated it by being frontline workers during the COVID-19 pandemic. Thus, the phrase “Nursing Now” has become more important than ever. The main aim of this article was to draw attention to the fact that 2020 was the Year of the Nurse and Midwife and, indeed, their role in the fight against the pandemic is difficult overlook. Through the use of available scientific databases, documents and scientific publications related to the subject were collected and analyzed. Nurses are able to fulfill their duties as long as they are properly rewarded and provided not only with support but also better terms and conditions of employment. The investment in nurses should also be treated as an investment in the healthcare system.

## 1. Introduction

Bearing in mind the contribution of nurses and midwives to the healthcare system as well as the 200th anniversary of Florence Nightingale’s birthday, the founder of modern nursing, the World Health Assembly announced the year 2020 to be the International Year of the Nurse and Midwife. This declaration took place in May 2019 at the 72nd Session of the World Health Organization’s (WHO) World Health Assembly (WHA) in Geneva, Switzerland, 20–28 May 2019. The ICN’s delegation, representing millions of nurses from around the world, had a high profile across the WHA’s agenda, intervening on several items to raise the nursing profession’s voice and highlight nursing’s presence. The announcement of the International Year of the Nurse and Midwife in 2020 was the first ever and a unique event to celebrate the benefits that nursing and midwifery bring to the health of the global population [1,2]. WHO representatives emphasized that this decision was based on the fact that this professional group accounts for more than half of all healthcare system employees and has always had an impact on the implementation of projects related to ensuring universal access to healthcare. Drawing attention to the role played by nurses in the healthcare system around the world is not a matter of coincidence but rather a reminder of the many years of their hard work and effort [3].

## 2. Materials and Methods

In order to implement this report, a review of available scientific literature and websites related to the subject of the work was conducted. The following search engines and databases were used: CINAHL, Web of Knowledge, Web of Science, PubMed Central, MEDLINE, Scopus, and Google Scholar.

In the search engines and databases, keywords and phrases were entered in accordance with the content and purpose of the work: “nurse”, “year of nurses and midwives”, and “nurse and COVID-19 pandemic”.

## 3. Findings

The Nursing Now campaign launched in 2016 as a result of the All-Party Parliamentary Group’s report showing the impact of nurses and midwives on improving health, promoting gender equality, and its correlation with supporting economic development. Another key evidence-based document was the 2019 publication of a white paper by the International Council of Nurses (ICN) and the Saudi Center for Patient Safety. This document brings together evidence from multiple sources, covering data from different countries, and clearly highlights the relationship between nursing and midwifery staff and patient safety. As indicated, only the appropriate number of nurses and midwives at the right time and in the right place is capable of ensuring the quality and safety of patients’ care. It is also a factor protecting the healthcare system against staff attrition in the profession due to the difficult working conditions, especially those related to insufficient nursing staff [4].

According to the WHO’s Director General, Tedros Adhanom Ghebreyesus, the declaration of 2020 as the Year of the Nurse and Midwife is a point of pride and honor and, above all, an opportunity to indicate the importance of the profession of nursing and midwifery to the interest of universal health protection around the world. This can also become, particularly in the face of a global shortage of nurses, a chance to make young people interested in this profession and encourage them to study in this field [5,6].

The actions taken also set the basis for the publication of the State of the World’s Nursing Report of 2020 by the WHO [7].

In 2020, many important events and inaugurations of Nursing Now campaigns were planned, which were to be honored by the presence of distinguished personalities in the world of science, culture as well as representatives of royal families. There were plans to organize numerous conferences, exhibitions, and meetings for this occasion as well as a roadshow throughout many regions for much of the year in Great Britain as well as in other countries. The activities that were to take place in 2020 were a turning point for changing the image of nursing at the local, national, and global levels and an opportunity to present one of the most trusted professions to the world [8,9,10].

All these ambitious plans were put to a stop by the news of a pandemic and a new virus threatening humanity. Subsequent reports on the growing number of infected individuals and the large efforts required to administer care of them were followed with disbelief. The COVID-19 pandemic has caught worldwide attention and never would all of the planning for the Year of the Nurse and Midwife celebrations resonate with such force and shine so brightly on the profession. It seemed that in the face of the horror of the pandemic, celebrating the Year of the Nurse and Midwife would be put aside, and no one would have the time or the strength to remember it. However, thanks to the courage and commitment of nurses and midwives, who suddenly found themselves on the frontlines in the fight against the COVID-19 pandemic, their special year began. Suddenly, the celebrations planned for the Nursing Now campaign proved to be a challenge, as the planned fanfare often turned into minutes of silence in tribute to deceased nurses [11]. Nurses and midwives worked despite insufficient human resources, without adequate protection in terms of personal protective equipment, sometimes in great decision-making and organizational chaos, and with a lack of knowledge about the new threat and methods of caring for COVID-19 patients. They are true heroes, torn between the concern for the welfare of patients’ families and their own, who have borne the enormous cost of this unequal struggle, namely, the loss of relatives, colleagues, or separation from close family members, children, or their sick parents in need of care. Pictures of nurses with wounds caused by scars from wearing masks and plastic coveralls were on the covers of magazines all over the world. To the nurses working despite a lack of sleep, dehydration, exhaustion, and longing for family, the hospital became both home and workplace. The nurses’ hands, although secured with rubber gloves, were the only ones that could be held by patients who were ill and dying alone [12,13,14].

In this way, the importance of this profession was recognized for the safety of all of us, and the year 2020 came about as a year of heroic effort made by nurses alike. The new situation did not put them off but brought about a great deal of goodness, patience, and the ability to do the work no man could do [14].

## 4. Discussion

The COVID-19 pandemic has claimed many lives, many of them medical personnel, nurses included. According to the World Health Organization, at least 115,000 health workers worldwide have died due to the COVID-19 pandemic, with the vast majority of them being nurses [15]. These severe losses might mean that patients, especially in the light of the global deficit in this profession, could be deprived of professional nursing care in many areas in the coming years. ICN data from 30 countries showed that, on average, 6% of all confirmed COVID-19 cases were healthcare professionals, with a range of 0–18%. If this percentage were to be repeated worldwide, 3.5 million confirmed COVID-19 cases would add to 210,000 infected healthcare workers [16]. According to available studies, 30% of those infected in China were healthcare workers, the vast majority of whom were nurses [17]. In Italy, 10% of medical workers became infected, and 3% of them died [18]. In the USA, over 3600 healthcare workers died during the first year of the pandemic, and 32% of them were nurses [19]. According to the WHO, at least 23,000 healthcare workers have been infected in more than 50 countries during COVID-19 pandemic, but there is no breakdown of how many of those were nurses [20]. While the whole world appreciates the work and sacrifice of nurses during the pandemic, it is astonishing and upsetting that there are no credible data on the rates of infection and death among nurses. The ICN calls on all governments to accurately record the number of nurses and other healthcare workers who have been infected with the virus or who died as a result of COVID-19 [16]. Perhaps, in many cases, these are losses that could have been avoided because they resulted from the lack of personal protective equipment (PPE), poor organization at work, or negligence on the part of decision makers [21,22,23,24]. According to Ali et al., healthcare workers are the frontline defense against COVID-19 infection among communities. Early evidence suggests that HCWs are being increasingly infected with COVID-19, ranging from 15% to 18% and, in some cases, up to 20% of the infected population [25].

A study conducted in Pakistan indicated that the lack of available appropriate PPE was the most significant factor responsible for the transmission of the COVID-19 virus among healthcare professionals [25]. An Italian study also pointed to a lack of adequate education among healthcare workers about the safe use of personal protective equipment and procedures to manage the COVID-19 pandemic [18]. The head of the International Council of Nurses said that, “the fact that as many nurses died during this pandemic as during World War I is shocking”, which is distressing and troublesome indicator. Catton stressed that this was not only the consequence of the current pandemic but the result of a lack of investment in nursing and the enormous shortage of nurses [17].

Like a phoenix from the ashes, nursing in the year 2020 shone with its own light that does not go out. This new situation, despite its gravity, also created development opportunities for many nurses. The pandemic restrictions meant that the use of telemedicine across the world has increased dramatically in 2020–2021. Solutions based on robotics and telemedicine have become widely used, enabling the remote care of patients and the ability to disinfect hospital rooms without the need to expose oneself to contact with COVID-19 [7,26]. A large-scale success was the implementation of the ICNP^®^ dictionary to medical entities and the introduction of the International Clinical Classification, providing opportunities to standardize the nursing practice in over 50 countries. This will, in turn, allow for the proper shaping of health policy around the world during a pandemic [27]. The necessity of carrying out mass vaccinations has resulted in the fact that in many countries, nurses obtained new qualifications allowing them to qualify patients for vaccination [28]. The main purpose of this article was to draw attention to the heroic work of nurses and the role they have to fulfil in society, especially during times of an epidemic.

## 5. Conclusions

As the International Council of Nurses President, Annette Kennedy, said on the occasion of the Integrated Delivery Network, “the COVID-19 pandemic has shown the world the role that nurses and midwives play in maintaining human health throughout life” [29]. The influence that nurses have on shaping health policy by working in all health sectors during the pandemic is a non-question. There is no doubt that the nurses can take care of one’s health, but they need investment as well as support and better terms and conditions of employment. This is the reason why, in the year 2021, the focus has been on the changes and innovations in nursing and how these will affect the future of healthcare [30]. The need to appreciate and put a strong emphasis on the work of nurses and other healthcare professionals was acknowledged by declaring the year 2021 the Year of Health and Care Workers by the World Health Organization, making it clear that it is high time to invest in health professionals, among whom nurses constitute the vast majority [31].

## Data Availability

The data presented in this study are available on reasonable request from the corresponding author.
